# Allograft Bone Screw in a Comminuted Hawkins III Talar Neck Fracture: Case Report

**DOI:** 10.3390/jcm13237457

**Published:** 2024-12-07

**Authors:** Konstanze Huetter, Patrick Holweg, Martin Ornig, Viktor Labmayr

**Affiliations:** Department of Orthopaedics and Trauma, Medical University of Graz, 8036 Graz, Austria; konstanze.huetter@medunigraz.at (K.H.); p.holweg@medunigraz.at (P.H.); martin.ornig@uniklinikum.kages.at (M.O.)

**Keywords:** allograft screw, defect bridging, talus fracture, Hawkins III, Shark Screw^®^, bone regeneration

## Abstract

**Background:** Talar neck fractures are complex injuries that become particularly challenging when accompanied by bone loss or comminution. This case report introduces the use of an allograft bone screw as a novel method for bridging lateral comminution at the talar neck, providing structural support and promoting bone regeneration. **Methods**: A 20-year-old male sustained a comminuted talar neck fracture with subtalar and tibiotalar dislocation after a bouldering fall. Urgent surgical intervention involved open reduction and internal fixation using a two-incision technique. The medial key fragment was stabilized with two conventional compression screws, revealing a significant lateral bony defect. This was bridged and stabilized with an allogeneic cortical bone screw (Shark Screw^®^, Surgebright GmbH, Lichtenberg, Austria), supplemented by bone replacement material. **Results**: At three months, CT confirmed fracture healing, and weight-bearing was initiated. At six months, the AOFAS score was 85/100 and FAAM 69/84, with no significant pain or swelling. By one year, the patient demonstrated full weight-bearing with occasional pain (AOFAS 88/100, FAAM 79/84). At two years, the patient achieved a pain-free range of motion and full activity participation (AOFAS 100/100, FAAM 83/84). **Conclusions**: The successful application of this technique illustrates the potential of allograft bone screws for stabilizing and bridging defects in talar neck fractures.

## 1. Introduction

Talus fractures are uncommon high-energy injuries induced by forced dorsiflexion with axial load [1]. The Hawkins classification aims to correlate fracture type to the risk of post-traumatic avascular necrosis (AVN), based on the talar blood supply and impact of perfusion disruption. Notably, a Hawkins type III talar fracture carries an AVN risk as up to 100%, and comminution of the talar neck compounds this risk [2,3,4]. Fixation strategies for talar fractures, particularly when comminution is present, must address the risk of instability and potential for collapse. In these cases, plate fixation is often employed to enhance structural integrity [5].

In its place, a novel human allogeneic cortical bone screw (Shark Screw^®^) was used to bridge a comminution zone and prevent valgus collapse. This allograft screw has primarily been used in osteotomies or simple fractures, e.g., scaphoid fractures [6,7,8]. To date, its concomitant use in acute trauma cases, particularly of this severity, has not been reported in the literature. Despite the significant risk of debility, at the two-year follow-up, the patient in this case was free of complaint.

## 2. Case Study

This case report was prepared following the CARE guidelines [9].

### 2.1. Patient Information, Clinical Findings and Diagnostic Assessment

A 20-year-old male presented at our level-1 trauma center in Austria following a fall during bouldering, resulting in an acute left ankle injury. He arrived via ambulance with the injured ankle stabilized in a vacuum splint. He reported no known allergies, medical prescriptions or relevant prior medical history, apart from recreational tobacco use. His body mass index (BMI) at admission was 29.3, classifying him as overweight.

**Figure 1:**
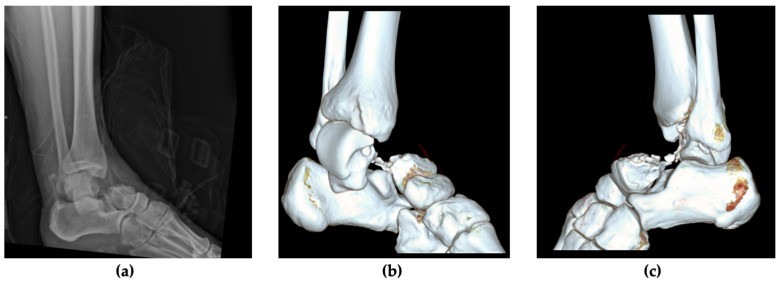
Radiological findings after immediate patient presentation in the vacuum splint. (**a**) Lateral view of the left ankle, showing a talar neck fracture with tibiotalar and subtalar dislocation; preoperative CT scans with 3D reconstruction: (**b**) medial view showing an extruded body of the talus; (**c**) lateral view showing a comminution zone at the lateral talar neck.

Upon initial examination, the patient exhibited a visibly deformed and painful left ankle joint. His skin integrity and peripheral motor and sensory functions were intact, though range of motion (ROM) was severely decreased. Prompt radiographic imaging assessed the extent of the injury, revealing a dislocated fracture of the talar neck. A CT scan revealed an extensive comminution zone in the lateral talar neck and confirmed a subtalar and tibiotalar dislocation. The talonavicular joint remained intact, classifying it as a Hawkins Type III talus fracture (Figure 1).

### 2.2. Therapeutic Intervention

In the emergency room, closed reduction was not possible. The patient was promptly taken to the operating room to undergo urgent surgical reduction and internal fixation. Under general anesthesia and following the administration of single-shot intravenous antibiotic prophylaxis, a two-incision technique utilizing an anteromedial and anterolateral approach to the talus was employed. Upon exposing the fracture, a significant comminution zone was identified, with a diastasis of approximately seven centimeters. Reduction was accomplished through traction combined with manual pressure applied to the dorsomedial aspect of the talus. The medial talar neck fracture was anatomically reconstructed with the key fragment and secured with K-wires to subsequently place countersunk cannulated compression screws (CCSs). A fragment of a fractured K-wire is visible on subsequent imaging.

**Figure 2:**
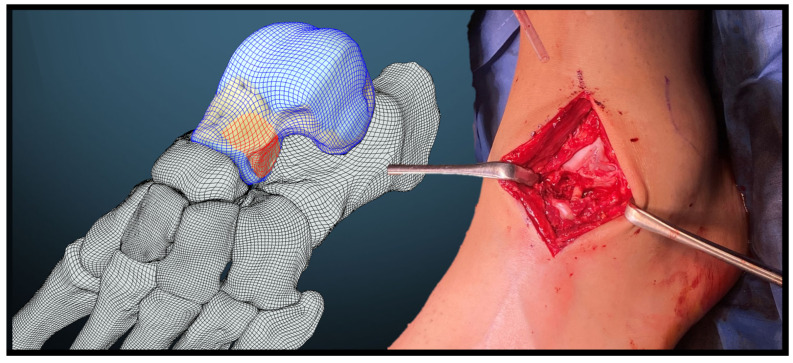
Diagram (**left**) of the hindfoot in lateral view, depicting the talus in a blue grid with the defect zone at the lateral talar neck highlighted in red. Intraoperative photograph (**right**) showing the anterolateral approach to the talus, illustrating the fracture and bone defect at the lateral talar neck. The allograft bone screw bridges and stabilizes the defect (note: bone replacement material Cerasorb® not yet in situ).

**Figure 3:**
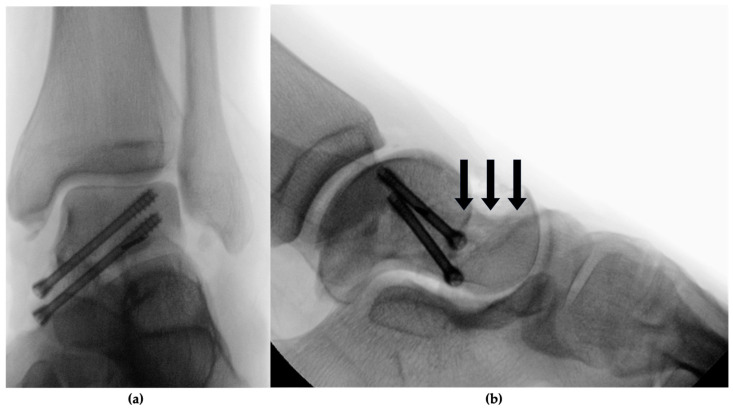
Intraoperative imaging following fracture reduction and screw fixation of the talus: (**a**) the AP view showing the two conventional screws (introduced medially), stabilizing the medial column, are visible on AP view and (**b**) lateral view. The allograft bone screw (lateral column) exhibits a similar radiolucency to the native bone; therefore, the position of the allograft bone screw is indicated by three arrows.

The lateral fracture zone, characterized by severe comminution, presented a bony defect (Figure 2). To address this, an allograft bone screw was utilized and combined with a bone replacement material composed of resorbable, pure-phase beta-tricalcium phosphate ceramic matrix (Cerasorb^®^, curasan AG, Kleinostheim, Germany). The defect was first bridged by over-drilling and inserting the allograft bone screw made from allogeneic human cortical bone, measuring 5 mm in diameter and 35 mm in length, and then filled with the bone replacement material. Intraoperative fluoroscopy was used to confirm reduction and fixation (Figure 3). The surgical procedure lasted 2 h and 34 min. Following the completion of the surgery, the left ankle was immobilized in a lower leg splint.

## 3. Follow-Up and Outcomes

The postoperative course was uneventful, and the patient was discharged on the fourth postoperative day in good general condition with a lower leg splint. Postoperative management included non-weight bearing and use of the splint for twelve weeks, with mobilization using crutches. Additionally, the patient was prescribed standard thrombosis prophylaxis with low-molecular-weight heparin, as-needed medication, and supplementation with vitamin D3 drops (cholecalciferol) were initiated. No early complications were observed, with satisfactory wound healing and intact neurovascular status.

**Figure 4:**
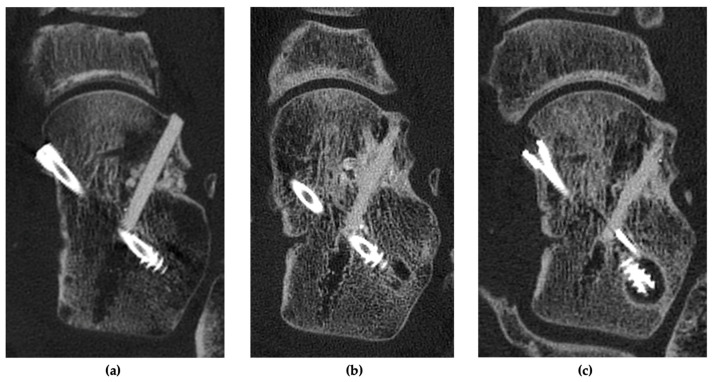
Axial CT scans of the reconstructed talar neck fracture with medially placed compression screws and laterally placed allograft bone screw with bone replacement material, showing the follow-up after three months (**a**), one year (**b**) and two years (**c**) with the osseointegration of the intact allograft bone screw without lateral column shortening (valgus collapse).

**Figure 5:**
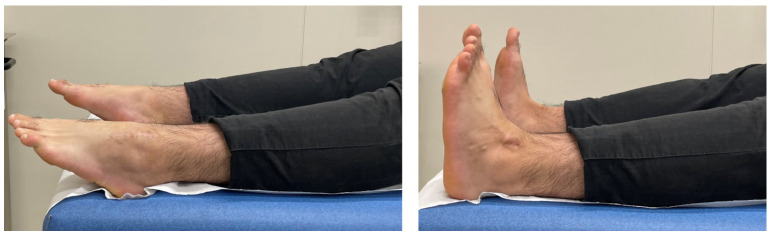
At the two-year follow-up, the patient demonstrated full range of motion of his left ankle with unrestricted plantarflexion (**left**) and dorsiflexion (**right**). A scar is seen on his left ankle.

**Figure 6:**
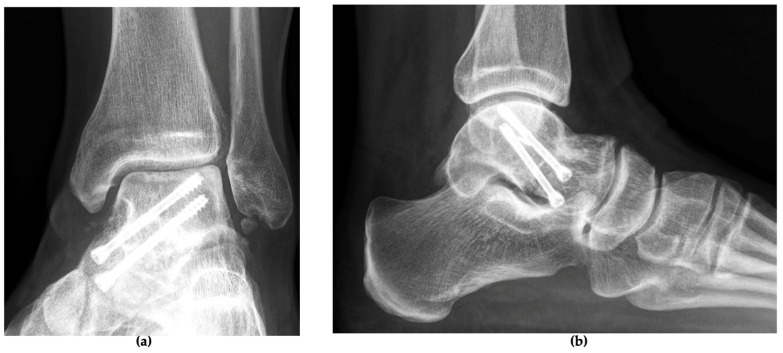
Radiographic imaging at the two-year follow-up in (**a**) AP view and (**b**) lateral view, showing a healed left ankle with restored anatomy.

At the three-month follow-up, the patient presented with low pain, yet persistent swelling of the left foot. During the physical examination, a dorsiflexion of 8 degrees was reached. Before mobilization, a CT scan was performed, which demonstrated progressive bony consolidation without implant failure (Figure 4). Therefore, weight-bearing was initiated. At the six-month follow-up, the patient achieved an American Orthopaedic Foot and Ankle Society (AOFAS) score of 85 out of 100 and a Foot and Ankle Ability Measure (FAAM) score of 69 out of 84 (82%), exhibiting no significant swelling or discomfort. At one year, the patient demonstrated full weight-bearing capabilities with occasional pain, resulting in an AOFAS score of 88 out of 100 and a FAAM score of 79 out of 84 (94%). A CT scan confirmed the integration of the allogeneic bone screw and the bone replacement material (Figure 4). At the final two-year follow-up, the patient reported a pain-free range of motion and full participation in activities (Figure 5), with excellent AOFAS and FAAM scores. (Table 1). No late complications were reported. A radiograph showed a healed talar neck fracture with anatomical restoration (Figure 6).

## 4. Discussion

Talar fractures, in general, are often linked to unfavorable postoperative outcomes [10]. It is therefore notable that this patient achieved complete recovery at the two-year follow-up, even reporting that there were times he almost forgot he had undergone surgery. This case is notable for the use of an allograft bone screw in the acute setting to manage a severe injury with reconstructive intent. This report details the application and outcome of this innovative approach.

As was used in this case, the standard approach for open reduction and internal fixation of displaced talus fractures involves using two incisions. This allows for better overview and control of the fracture [11]. Methods of open reduction and internal fixation range from screws to plating or bone grafting. In a displaced talar neck fracture, compression screws placed in a sagittal plane might hold the fracture mechanically, but when paired with a comminution zone, could lead to talar neck shortening. Therefore, plating for defect stabilization (whether medial, lateral or bilateral) has been shown to reduce the risk of over-compression or collapse [12]. A standard technique is the use of a mini-fragment buttress plate as a supplementation to screw fixation [13]. As an alternative, we used an allograft bone screw with bone replacement material for primary reconstruction. This minimizes the risk of mechanical irritation and the subsequent hardware removal.

Bone grafting has been reported in the literature, usually for large impaction defects of the medial talar neck [1]. There is the option of harvesting an autologous bone graft, e.g., from the iliac crest [14]. However, this technique requires a longer surgery time and a separate approach, with its own general surgical risks of wound healing disorder, infection, or scarring. While it has been recommended to rely on plate fixation for securing a cancellous bone graft in place [15], there have not been methods to bridge a defect with allografts and hold it in place at once: the novelty of the allograft bone screw lies in its dual function by providing both stability and biological integration in a single method [16].

A Hawkins type III talar neck fracture is at exceptional risk of perfusion disruption of the talar bone, increasing the risk for AVN [1]. It can be argued that the osteoconductive properties of the allograft bone screw may be beneficial in avoiding such a complication. When placed in vital bone, it connects to the surrounding blood supply, allowing host cells to grow into the Haversian canals within the allograft bone screw [17]. Furthermore, a frequent post-traumatic sequelae is malunion and subsequent arthrosis, particularly as the degree of displacement increases [18]. Therefore, the fixation aims primarily at achieving joint surface realignment and reconstructing the medial and lateral column lengths. This reconstructive osteosynthesis is pivotal in order to prevent talar neck shortening and valgus collapse [19,20].

This aim is in line with the surgical approach used in this case, where two medial and two countersunk compression screws held the medial column in place, and the allograft bone screw stabilized the lateral column.

This case study is subject to limitations inherent to its nature, as a single case may limit the generalizability of its findings. While the AOFAS and FAAM scores provide objective data, there is a potential for subjective reporting, for example, in the assessment of daily pain.

Acknowledging these limitations, this case report illustrates a detailed clinical presentation with standardized scoring systems in a longitudinal follow-up. It therefore makes a strong case for the potential of using an allograft bone screw in patients with comminuted fracture zones; however, due to the scarcity of available literature on bone grafting, further high-level evidence studies are needed to confirm our findings.

## 5. Conclusions

This case introduces the use of an allograft bone screw to stabilize the lateral column and bridge a complex defect in a severe Hawkins type III talar neck fracture. Providing both structural stability and, in combination with bone replacement material, a scaffold for bone regeneration, this approach achieved full anatomical reconstruction and complete clinical recovery. This technique offers a promising addition to the surgical options available for complex talar fractures.

## Figures and Tables

**Table 1:** Timeline with scores during the follow-up of two years.

	6 Months	1 Year	2 Years
AOFAS	85/100	88/100	100/100
FAAM	69/84 (82%)	79/84 (94%)	83/84 (99%)

## Data Availability

The original contributions presented in this case study are included in the article, and further inquiries can be directed to the corresponding author.
